# The risks of self-made diets: the case of an amateur bodybuilder

**DOI:** 10.1186/s12970-015-0077-8

**Published:** 2015-04-01

**Authors:** Lucio Della Guardia, Maurizio Cavallaro, Hellas Cena

**Affiliations:** Department of Public Health, Experimental and Forensic Medicine, Unit of Human Nutrition, University of Pavia, via Bassi 21, 27100 Pavia, Italy

**Keywords:** Supplementation, Vitamin and micronutrients overdose, Gastrointestinal, High-protein diet, Bodybuilding, DIY diets

## Abstract

**Background:**

Following DIY (do it yourself) diets as well as consuming supplements exceeding by far the recommended daily intake levels, is common among athletes; these dietary habits often lead to an overconsumption of some macro and/or micronutrients, exposing athletes to potential health risks.

The aim of this study is to document the development of possible adverse effects in a 33 year-old amateur bodybuilder who consumed for 16 years a DIY high protein diet associated to nutrient supplementation. Body composition, biochemical measures and anamnestic findings were evaluated.

We present this case to put on alert about the possible risks of such behavior repeated over time, focusing on the adverse gastrointestinal effects. We discuss the energy and nutrient composition of his DIY diet as well as the use of supplements.

**Conclusion:**

This study provides preliminary data of the potential risks of a long-term DIY dietary supplementation and a high protein diet. In this case, permanent abdominal discomfort was evidenced in an amateur body builder with an intake exceeding tolerable upper limit for vitamin A, selenium and zinc, according to our national and updated recommendations.

As many amateur athletes usually adopt self-made diets and supplementation, it would be advisable for them to be supervised in order to prevent health risks due to a long-term DIY diet and over-supplementation.

## Background

Nutritional supplements are commonly used by elite and amateur athletes. Supplements are often considered necessary to maintain strength as well as to enhance endurance performance and to improve the ability to train longer [1].

It is estimated that up to 90 percent of all the athletes globally use supplements to some extent [2] mainly because these substances are freely available to purchase. In particular, amateur athletes frequently follow their own prescriptions despite the recommendations regarding the risks connected with prolonged and excessive intake of specific nutrients [3-5]. Indeed, although various nutrients are required for normal growth, maintenance and repair of tissues, it has been demonstrated that the excess of nutrient intake may cause adverse effects on organs and metabolism [3-5].

Furthermore, there is a substantial risk of supplements contamination with prohibited substances such as stimulants or hormone-like compounds [6,7].

Whether a scheduled supplementation of some and specific macronutrient compounds could enhance muscle adaptation to training [8], no precise reason, even among athletes, seems to justify a massive intake of some nutrients, especially vitamins and minerals, if the dietary regimen provides for a sufficient variety of foods [9]. Therefore, many efforts have been performed to identify macro and micronutrient intake ranges of safety.

The purpose of this study is to document the development of possible adverse effects in an amateur bodybuilder on a long term DIY high protein diet associated to nutrient supplementation.

## Case presentation

According to the international dietary guidelines [10] and the dietary reference intakes (IOR)^a^ [11] a balanced diet should respect some paradigms regarding the macro and micronutrient intake. In detail, the guidelines specify that carbohydrate (CHO), lipids and protein intake should represent specific percentages of the total energy intake (CHO 55%, lipids less than 30% and protein around 15%) [11,12]. Although, as far as protein intake is concerned, the daily-recommended amount should be related to the body weight (0.9 g of protein/kg/day) [11,12].

In case of trained athletes, the protein intake can be increased to 1.2-1.7 g/kg/day, in order to satisfy the augmented muscle turnover depending on the type and amount of sport activity [9].

Fiber intake is recommended in a quantity of 20–30 g/day in order to maintain a correct gastrointestinal activity and mucosal tropism.

Moreover, recommendations of daily vitamins and minerals intakes have been provided in order to satisfy the body metabolic request and to avoid toxicity [12].

Amateur athletes often go on do-it-yourself (DIY) diets that differ somewhat from the above recommendations and may lead to health hazards.

We discuss the development of possible adverse effects in a 33 year-old amateur bodybuilder on a long term DIY high protein diet associated to micronutrient supplementation, referred to our department by his personal trainer after reporting deep weakness and recurrent episodes of diarrhea in the previous 6 months.

The patient was a warehouse worker. He used to follow a DIY diet along with a multivitamin and mineral supplementation.

### Symptoms

The patient complained of feeling fatigue and tiredness, interfering with his work activity as well as with his training and performance. The anamnestic interview did not record any insomnia episodes or other sleep disturbances. The sleep period was around the 6 hours per night. Moreover, the patient described a strong gastrointestinal discomfort such as post-prandial fullness, nausea and dyspepsia; the main symptoms reported by the patient were frequent episodes of diarrhea that occurred around 3 times per day, minutes to hours after the ingestion of food, followed by moderate-to-intense low abdominal pain. Diarrhea and the gastrointestinal distress reported had occurred approximately for 6 months before the patient underwent our medical examination.

### Anthropometric and nutritional evaluation

We conducted a complete nutritional evaluation. The medical inspection included: physical examination, blood tests recording, anthropometric measurements, nutritional and medical history collection, body composition analysis by bioelectrical-impedance (BIA-101 model; Akern srl, Florence, Italy) and resting energy expenditure by indirect calorimetry (Vmax Spectra 29n; Sensormedics, Yorba Linda, California, US). The last two tests were conducted under standard conditions: the patient was dressed in light clothing and the average room temperature was around 21°C; the patient had been advised to fast for 12 hours, abstain from alcohol consumption and refrain from any physical activity in the 24 hours before the measurements.

Heart rate and blood pressure were in the normal range (61 bpm and 125/80 mmHg); no signs of respiratory, skin or mucosal alterations were reported. Notably, the patient felt pain in epigastrium and in the right iliac fossa during abdominal palpation.

Height (m 1.86) and weight (86.3 kg) of the subject were measured and recorded in nearest 0.1 and 1 cm, respectively. BMI (24.9 kg/m^2^) was then calculated dividing the weight (kg) by the height in square meters (m^2^).

Body composition by bioelectrical-impedance analysis, revealed a moderate-to-high percentage of metabolically active mass (body cellular mass 48.5%, body fat-free mass 79.9%, body fat mass 20.1%, phase angle 6.8°)^b^.

Anthropometric, body composition and blood pressure characteristics are reported in Table 1.

**Table 1:** **Patient’s characteristics**

**Variable**	**Value**
Height (m)	1.86
Weight (kg)	86.3
BMI (kg/m^2^)	24.9
WC (cm)	89
SBP/DBP (mmHg)	120/80
Arm circumference (cm)	33
Fat mass kg (%)	17.3 (20.1)
Fat-free mass kg (%)	69 (79.9)
Body cellular mass kg (%)	33.5 (48.5)
Extracellular water kg (%)	19.1 (38.5)

Indirect calorimetry measured a respiratory quotient of 0.9 and a resting energy expenditure (REE) of 1554 kcal/day, equivalent to the 79% of the estimated value according to Harris-Benedict equation [13]. This disparity was probably due both to the complete suspension of rigorous training (for a 6 months period) than to the self-induced food restriction in the effort to try to manage the gastrointestinal discomfort. The prolonged training suspension period was likely to lead to a muscle mass loss and relative increase of fat mass [14]. Besides, the low resting energy expenditure observed is likely to reflect the body-self adjustment secondary to the scarce physical activity performed as well as the spontaneously decreased energy daily intake [12,15].

Unfortunately, not in possession of the body composition data prior the 6-month suspension of training, we are not able to comment in more detail the above-reported findings.

### Laboratory analysis

All measured biochemical parameters (blood count as well as serum electrolytes, total protein, hepatic markers, ferritin, creatinine, C-reactive protein, pancreatic enzymes and insulin) were within the reference values. The patient also underwent a cardiological assessment (electrocardiography and echocardiography) that did not show any alteration. The fecal fat test identified a slight fat malabsorption. Markers of celiac disease, Helicobacter Pylori antibodies and parasite exam were all negative. The total IgE value resulted within the range . The allergen-specific IgE assay showed no positivity for the food allergens tested. The H_2_ breath test showed lactose intolerance. Basal biochemical data are shown in Table 2.

**Table 2:** **Patient’s biochemical data**

**Metabolite**	**Value**
Urea nitrogenum (mg/dl)	47
Creatinine (mg/dl)	0.92
Total protein (g)	8.0
Fasting glucose (mg/dl)	69
TSH (UI/l)	1.17
Ca^2+^ (mg/dl)	10.1
Na^+^/K^+^ (mEq/l)	139/3.8
Helicobacter Pilory IgG	Negative
Anti-gliadine IgA-IgG	Negative
H_2_ breath test	Positive
Fecal occult blood test	Negative
Basal insulin (mUI/l)	14
Fecal simple sugars (g/l)	1.8
Fecal fats (g/dl)	2.2
Total IgE (UI/ml)	110

The oral glucose tolerance test revealed impaired glucose tolerance (glycaemia: 153 mg/dl after 2 hours). The esophagogastroduodenoscopy performed did not display any remarkable anatomic alteration.

### Dietary data

The diet history collected by trained nutrition professionals included a detailed interview about usual pattern of eating, a food list asking for amount and frequency usually eaten, and a 7-day dietary record.

The major strength of the diet history method is its assessment of meal patterns and details of food intake rather than intakes for a short period of time. We used this approach since our patient had been following a specific DIY eating pattern and this method is a tool able to ascertain the usual eating patterns for an extended period of time, including type, frequency and amount of foods consumed [16].

Portion sizes were estimated through a validated colour food photography atlas for quantifying the portion size eaten [17].

Supplement use, frequency and dosage were investigated.

According to the 7-day food diary compiled by the patient and the 24-h recall performed, the patient’s daily energy intake, at the time of the medical examination, was approximately 2160 kcal/day (Table 3). Furthermore, the vitamin and mineral supplementation, he reported he had never stopped, exceeded by far the micro nutrients intake recommended by the Italian Official Reccomendations (IOR) [11] (Table 4).

**Table 3:** **Dietary intake at the time of the medical examination (7-day food diary assessment)**

**Component**	**Quantity**	**% of TE**	**IOR** ^**§**^
Energy (kcal)	2160	-	-
Total protein (g)	106.9	19.7	-
Total fats (g)	46.1	19.2	20-35%
*Saturated fats (g)*	9.1	3.8	<10%
*MUFA (g)*	24.8	10.3	10-15%
*PUFA (g)*	9.4	3.9	5-10%
Total carbohydrates (g)	352	61.1	45-60%
*Starch (g)*	297	51.5	45-53%
*Simple sugars (g)*	55	9.5	<15%
Dietary fiber (g)	16	-	12.6-16.7*
Iron (mg)	15	-	10
Cholesterol (mg)	212	-	0-300
Vitamin A (RE) (μg)	174	-	700
Niacin (mg)	35	-	18
Selenium (μg)	25	-	55
Zinc (mg)	14	-	12

% of TE: percentage of total energy intake.

IOR: Italian official recommendations reference values.

^§^IOR reference values are reported as % of TE for macronutrient and as daily total amount for micronutrient and fiber.

*g/1000 kcal of energy intake.

**Table 4:** **Reference values and content of vitamins and minerals per tablet of supplement (1 tablet/day)**

**Vitamin**	**IOR (per day)**	**UL (per day)**	MPL*	**TC**
Vitamin A (μg)	700**	3000	1200	6000
Vitamin D (μg)	15	100	25	10
Vitamin E (mg)	13	300	60	66.7
Vitamin K (μg)	140	-	105	-
Vitamin C (mg)	105	-	1000	150
Thiamin (mg)	1.2	-	25	25
Riboflavin (mg)	1,6	-	25	25
Niacin (mg)	18	900^§^	36	100
Vitamin B6 (mg)	1,3	25	9,5	25
Folic acid (μg)	400	1000	400	800
Vitamin B12 (μg)	2,4	-	33	100
Biotin (μg)	-	-	0.450	300
**Minerals**				
Calcium (mg)	1000	2500	1200	25
Magnesium (mg)	240	250	450	7.2
Iron (mg)	10	-	30	10
Zinc (mg)	12	25	12.5	15
Copper (mg)	0.9	5	2	2
Manganese (mg)	2.7	-	10	5
Selenium (μg)	55	300	83	200

IOR: Italian official recommendations.

MPL: maximum permitted level in supplements (per unit of dosage).

TC: content per table of supplement.

UL: tolerable upper levels of intake.

*Maximum daily dosage of supplement allowed according to Italian Ministry of Health.

^§^Vitamin A is reported as Retinol Activity Equivalents.

Data about the remote dietary history were collected. The DIY dietary scheme, handed out by the subject, was then analyzed for energy, macro and micronutrient content and compared to the dietary intakes recommended by the IOR [11] using a software with BDA food composition tables [18] as database.

The results showed that the patient usual dietary intake for up to 6 months before was very high in protein and poor in fiber; protein daily intake, mainly from animal-sources, was around 2.3 g/kg/day (Table 5). Moreover, in the same period, the patient used to train about 5 times a week, also supplementing his diet with milk-derived protein drinks (30 g of whey protein/day, right after each training session), along with multivitamin and mineral supplementation. According to the data collected, the patient’s mean daily energy intake was about 3000 kcal/day (Table 5). The usual daily water intake was up to 5 liters per day. The patient has not been consuming coffee, tea, fructose or sweeteners of any type.

**Table 5:** Composition of the DIY dietary scheme followed during the last 16 years * (except for the last 6 months)

**Component**	**Quantity**	**% of TE**	**IOR** ^**§**^
Energy (kcal)	2967	-	-
Total protein (g)	199	26.8	-
Total fats (g)	74	22.5	20-35%
*Saturated fats (g)*	17	5.2	<10%
*MUFA (g)*	44	13.6	10-15%
*PUFA (g)*	8	2.4	5-10%
Total carbohydrates (g)	401	50.7	45-60%
*Starch (g)*	316	39.9	45-53%
*Simple sugars (g)*	85	10.7	<15%
Dietary fiber (g)	19	-	12.6-16.7**
Iron (mg)	20	-	10
Cholesterol (mg)	493	-	0-300
Vitamin A (RE) (μg)	1087	-	700
Niacin (mg)	71	-	18
Selenium (μg)	88	-	55
Zinc (mg)	19	-	12

% of TE: percentage of total energy intake.

IOR: Italian official recommendations reference values.

^§^IOR reference values are reported as % of TE for macronutrient and as daily total amount for micronutrient and fiber.

*Milk-protein supplementation is not reported in the list.

**g/1000 kcal of energy intake.

He reported to have followed the same dietary pattern for 16 years with no adverse effects.

Six months before our medical nutrition evaluation, the patient switched to a lactose-free diet, suspending the milk-derived protein supplements as well, since he stated he had developed lactose intolerance symptomatology.

At the time of the medical examination, the patient had not yet resumed workouts, suspended 6 months before, due to persistent tiredness, gastrointestinal discomfort and mood deflection.

### Dietary therapy

We estimated the daily energy expenditure multiplying REE measured by indirect calorimetry to physical activity level (PAL = 1.65)^c^ [19] and therefore developed a physiological dietary plan meeting the actual energy, macro and micronutrient requirements (Table 6). The dietary plan was aimed primarily to establish a correct energy intake in order to counteract the feeling of weakness and to correct inappropriate eating habits. It was also paid particular attention to those nutrients possibly implicated in the development of gastrointestinal distress (simple sugars, fats, fiber). In addition, in order to rebalance the intestinal microflora and improve the enteric trophism reducing diarrhea and GI discomfort we prescribed a multiple strain probiotic with a mixture of prebiotics and antioxidants vitamins especially useful in intestinal dysbiosis and inflammations [20,21]. To this was added a EPA /DHA and Vitamin E supplement (300 mg/200 mg and 1.8 mg per tablet) for 2 weeks to further reduce the gastrointestinal inflammation [11,15].

**Table 6:** **Dietary composition of the nutritional therapy prescribed**

**Component**	**Quantity**	**% of TE**	**IOR** ^**§**^
Energy (kcal)	2601	-	-
Total protein (g)	122	18.8	-
Total fats (g)	71	24.5	25-35%
*Saturated fats (g)*	13	4.5	<10%
*MUFA (g)*	44	15.2	10-15%
*PUFA (g)*	11	3.8	5-10%
Total carbohydrates (g)	393	56.6	45-60%
*Starch (g)*	343	49.4	45-53%
*Simple sugars (g)*	50	7.2	<15%
Dietary fiber (g)	29	-	12.6-16.7*
Iron (mg)	19	-	10
Cholesterol (mg)	207	-	0-300
Vitamin A (RE) (μg)	835	-	700
Niacin (mg)	27	-	18
Selenium (μg)	35	-	55
Zinc (mg)	14	-	12

% of TE: percentage of total energy.

IOR: Italian official recommendations reference values.

^§^IOR reference values are reported as % of TE for macronutrient and as daily total amount for micronutrient and fiber.

*g/1000 kcal of energy intake.

Finally we encouraged the patient to suspend the multivitamin and mineral supplementation and to restrain from taking protein supplements.

The follow-up visit conducted 1 month later reported a slight improvement of the gastric post-prandial discomfort and the diarrhea episodes frequency reduction. The improvement of gastrointestinal symptoms allowed the patient to increase the daily energy intake as assessed by the 24 h recall method.

## Discussion

A resistance athlete’s diet needs to be different in macronutrient and in micronutrient composition compared to a normally active subject’s diet. For a resistance athlete it should be considered a higher daily protein requirement in order to satisfy the muscle accretion needs as well as the muscle protein synthesis increase [8,9]. Protein intake should be increased to 1.2-1.7 g/kg/day, enough to ensure the increased muscle needs [9]. Besides proteins, CHO intake and remarkably its timing, seems to be important for the muscle accretion and weight lift performance of these athletes. The reference range is 8–10 g/kg/day of CHO [8,22].

Although exercise leads to an oxidative stress increase and loss of some minerals, there are no evidences justifying the massive intake of vitamin-mineral supplements, especially among amateur athletes [9,22]. Even considering the hypothetical augmented needs of a bodybuilder, in our case-report, the daily intake of some nutrients was far higher than advisable. According to the data we obtained from the dietary scheme he handed us out and the nutritional anamnesis we collected, the patient’s dietary pattern differs somewhat from the official guidelines [10]: quite higher in protein, vitamins and minerals intake than recommended [11,12]. The diet history collected showed a 16-year period of large dietary protein intake (approximately around the 2.5 g/kg/day, also considering the extra protein supplementation) mostly from animal sources. It is conceivable that the long period of unsupervised supplementation and self-made diet consumption may have led to the development of adverse effects. It is not uncommon that the large fortified food consumption, associated with supplements, may lead to an excessive intake of vitamins and minerals that come close to or exceed the Tolerable Upper Intake Level (UL).

Indeed, many of the side effects exerted by the supplements are related to the gastrointestinal tract [3].

The patient reported the continuative and habitual intake of milk-derived protein supplements. Although the metabolic side effects of a high protein diet is not completely clarified, it is possible that the prolonged high protein load may have induced a slight bowel mucosal dysfunction; some bacterial protein-derived metabolites such as ammonia and short-chain fatty acids are likely to interfere with the colonic epithelium metabolism and physiology [23]; ammonia at millimolar concentrations in the bowel lumen has been shown to exert deleterious effects on the colonic epithelium [24] and to alter short-chain fatty acids oxidation in isolated colonocytes [25]. Interestingly, recent research has reported [26] that feeding mice with a very high protein diet causes the development of peculiar alterations in the enterocytes metabolism, such as t the mucosa thinning and an overall impairment in water absorption.

In our case, we noticed that more than five components listed in the supplement nutritional label, exceeded the IOR as well as the Maximum Permitted Level in supplements (MPL) in Italy [27]. Therefore, given that the patient has been exposed to a high level of micronutrients intake, consuming many fortified foods apart from supplements, it seems reasonable that the overall excessive micronutrient intake may partially explain the reported symptomatology.

In particular Vitamin A, Niacin, Zinc and Selenium overdose intake will be discussed.

*Vitamin A* is an essential component for human growth, gene expression and immune system. Vitamin A includes a family of fat-soluble molecules such as retinol and pro-vitamins A carotenoids (mainly β-carotene). The RDA for men is around the 900 μg/day of retinol activity equivalents (RE) while the UL for adults is set at 3000 μg/day of preformed vitamin [11,28]. Notably, the recent IOR for men is 700 μg of RE/day [11,3]. Excessive prolonged intake of vitamin A (months or years) may produce toxicity with symptoms such as nausea, vomiting and diarrhea [4]. The patient’s daily intake of Vitamin A as a supplement (β-carotene and retinyl palmitate, in 1 tablet of supplement) was 6000 μg of RE. The whole dietary daily intake (dietary + supplementation) had been around the 7080 μg of RE for several years.

*Niacin*, as a precursor of nicotinamide, plays its biological role in many reactions connected to the energy production. In the form of nicotinic acid it is employed to treat different types of dyslipidemia and to lower overall risk of developing atherosclerosis [29,30]. Its recommended intake in adult males is be around the 18 mg/day and should not exceed 30–35 mg/day (which corresponds to 900 mg/day of nicotinamide and 10 mg/day of nicotinic acid) [11,31]. In our case report, the overall niacin daily intake (mean dietary intake plus supplement) was around the 170 mg/day. Although the common side effects reported by niacin overconsumption are usually related to skin flushing (niacin intake from 30 to 1000 mg/day) and hepatotoxicity as shown in several studies [5,31], niacin “overdose” might also be related to gastrointestinal discomfort, as already reported for doses > 2000 mg with subsequent episodes of severe diarrhea and/or transaminase increase [31]. The development or exacerbation of peptic ulcer as well as nausea and vomiting have been described by several authors, in cases of very high doses of niacin intake during anti-atherosclerotic therapy [5,31].

*Zinc* is another nutrient presumably involved in the development or exacerbation of abdominal discomfort. The mechanism of damage could be related to a direct corrosive action of the metal on the mucosal wall, after reacting with the gastric secretion [32]. The recommended zinc intake should be in the range of 8–13 mg/day, without exceeding 25 mg/day [11,30]. Higher zinc intake might promote nausea, abdominal cramping, vomiting, and diarrhea [32-34]. In our case, this micronutrient intake (dietary intake plus supplementation) was about 34 mg/day. High doses of zinc sulfate, as those employed in the treatment of Wilson’s disease, have been associated to the development of some gastrointestinal disorders such as dyspepsia, vomiting, nausea and loss of appetite [35].

*Selenium* is an important co-factor for several biological molecules and enzymes playing an important role in redox reactions and hormone production. The currently recommended safety range intake for male adults is less than the 400 μg/day, while the IOR UL is set at 300 μg/day [11,36]. The patient’s overall intake was about 288 μg/day. A higher intake is likely to lead to the toxic effects on the endocrine function as well as on the gastrointestinal tract [37]. In 2008, a poorly manufactured multivitamin was responsible for more than 200 cases of selenium poisoning with symptoms including diarrhea, fatigue, hair loss, and joint pain [38].

Moreover, the development of gastrointestinal adverse effect could also be interpreted considering the possible presence of contaminating compounds within the supplements. Many supplements have been found to be adulterated with pharmaceuticals or pharmaceutical analogues, including stimulants, anabolic steroids, antidepressants, psychotropic substances [39,40]. Stimulants and doping compounds [7,41], as well as new analogue of amphetamine, have been found in widespread sport supplements [7]. Also the above-mentioned selenium contamination of multivitamin supplement was considered a case of supplementation poisoning [38]. Other studies pointed out the presence, frequently, of compounds with 17β-estradiol-like activity or GHPR-2 in these supplements, which may interfere with some key points of the hormonal metabolism [11,42]. The presence of such hidden substances may determine the development of side effects that go beyond simple constituents of the supplement-in-itself, especially when the supplementation is carried out with more than a single supplement and consumed every day for years.

The fecal test (mucus, blood, erythrocytes, leucocytes) and all the biochemical parameters connected with a possible IBD (Hb, leucocytes, RCP) resulted in the normal range. The external medical inspection also led to rule out an IBD in existence. The gastroscopy performed and the negative result of celiac antibodies confuted any diagnostic suspect of celiac disease. No history of inhaled or food allergy was recorded. The value of the total IgE was in a normal range. The specific IgE test performed on different food allergens showed no positivity. The last findings led us to rule out the presence of food allergies. Although the patient resulted lactose intolerant, we excluded that it could be connected with the symptoms suffered because since he developed the lactose intolerance he self-suspended any milk and milk-derived food consumption as well as supplementation with milk derived proteins, without achieving any amelioration of the gastrointestinal distress. The low amount of simple sugars introduced (Tables 3 and 5) as well as the result from fecal analysis led us to rule out any possibility of osmotic diarrhea. After excluding this common causes of abdominal distress and diarrhea, we infer that the gastrointestinal symptoms reported by this patient of ours such as osmotic diarrhea, abdominal cramping and/or nausea might be linked to the poor quality of his dietary pattern and in particular to the massive intake of protein and micronutrients, secondary to suboptimal diet and supplementation [22-26,29-42]. Although we believe that our patient’s clinical situation did not completely match for the established criteria of diagnosis of irritable bowel syndrome (IBS), we cannot completely rule it out. The low mood, the low visceral pain threshold and the symptomatology reported lead to suppose the presence of an IBS as well. Actually, given that the IBS is a nosological status with a complex and not well-defined pathogenesis, we are likely to believe that the IBS could have been triggered or possibly enhanced by the patient’s suboptimal diet. The glucose intolerance detected still remains an unsolved question. The basal insulin and HOMA index were in a normal range. The fasting glucose level was even slightly lower than recommended. To partially explain the findings we supposed that the GI distress and the patent’s whole clinical situation were likely to determine an increase in stress hormone levels (adrenaline, cortisol). As these hormones play a diabetogenic effect, we can assume that the detected glucose intolerance may be thus explained.

The main limitation of this study is related to the short period of follow-up carried out (the results are related to 1 month therapy) due to the poor compliance of the patient to the new dietary scheme and recommendations. We are likely to believe that the low mood and the vicious circle induced by the GI discomfort, the low training level and the partial disappearance of the symptoms played an important role in the dropout. Furthermore, the patient did not willingly accept the indication to the micronutrient supplementation suspension, which he considered necessary to cover his metabolic needs. The follow-up brevity and the patient’s scarce compliance to the therapy did not allow us to obtain any further data to confirm or disprove our diagnostic hypothesis. Another limit regards the lack of any data about his physical condition or muscular strength in order to prove the basal performance and the improvement after our nutritional intervention. Similarly, we were unable to report any training log, which would have been helpful to better understand the clinical situation. Finally, the lack of epidemiological and human studies useful to confirm our hypothesis or contradict it, the absence of significant similar reports in literature, the atypical clinical presentation and the differential diagnosis make our hypothesis speculative and the case difficult to interpret but worthy of being reported.

## Conclusion

The present study highlights the risk of adverse effects in prolonged suboptimal macro and micronutrient supplementation in a subject involved in non-competitive sport.

As showed in this case, the common practice of adopting “self-made” nutritional regimes among amateur athletes may lead to an unusual gastrointestinal symptomatology. Moreover, future chronic complications cannot be completely ruled out. Although many amateur bodybuilders believe that “more is better” underestimate the importance of diet and overestimate the effects of supplements, unaware of “over supplementation” side effects.

In order to prevent severe and chronic health effects, nutritionists and trainers should advise the athletes about the possible risks of self-made diets that provide for extra supplementation, especially when carried out for long time.

## Consent

Written informed consent was obtained from the patient for publication of this Case Report. A copy of the written consent is available for review by the Editor-in-Chief of this journal.

## Endnotes

^a^The considered IOR are referred to adult males aged between 30–59 y-old.

^b^Body fat mass and fat-free mass were estimated by BODYGRAM software (AKERN, Florence, Italy).

^c^The 1.65 value of PAL has been chosen considering a light level of physical activity in accordance to the WHO/FAO/UNU guidelines.

## Abbreviations

IOR: Italian official recommendations
DIY: Do it yourself
BIA: Bioelectrical-impedance
MPL: Maximum permitted in supplements
RE: Retinol activity equivalents
UL: Tolerable upper intake level
TC: content per tablet of supplement
